# Implicit Motives and Men’s Perceived Constraint in Fatherhood

**DOI:** 10.3389/fpsyg.2016.01856

**Published:** 2016-11-23

**Authors:** Jessica Ruppen, Patricia Waldvogel, Ulrike Ehlert

**Affiliations:** ^1^Clinical Psychology and Psychotherapy, Institute of Psychology, University of ZurichZurich, Switzerland; ^2^Central European Network on Fatherhood, Headquarter at University of ViennaVienna, Austria; ^3^University Research Priority Program – Dynamics of Healthy Aging, University of ZurichZurich, Switzerland

**Keywords:** implicit motivation, affiliation, power, fatherhood, perceived constraint, life satisfaction

## Abstract

Research shows that implicit motives influence social relationships. However, little is known about their role in fatherhood and, particularly, how men experience their paternal role. Therefore, this study examined the association of implicit motives and fathers’ perceived constraint due to fatherhood. Furthermore, we explored their relation to fathers’ life satisfaction. Participants were fathers with biological children (*N* = 276). They were asked to write picture stories, which were then coded for implicit affiliation and power motives. Perceived constraint and life satisfaction were assessed on a visual analog scale. A higher implicit need for affiliation was significantly associated with lower perceived constraint, whereas the implicit need for power had the opposite effect. Perceived constraint had a negative influence on life satisfaction. Structural equation modeling revealed significant indirect effects of implicit affiliation and power motives on life satisfaction mediated by perceived constraint. Our findings indicate that men with a higher implicit need for affiliation experience less constraint due to fatherhood, resulting in higher life satisfaction. The implicit need for power, however, results in more perceived constraint and is related to decreased life satisfaction.

## Introduction

Fatherhood is an important developmental life phase for men. However, the paternal role can be experienced as both constraining or fulfilling and therefore, may have both positive and negative consequences for men’s well-being and satisfaction with life. Despite the importance of this topic, little is known about men’s subjective experience in fatherhood. Moreover, factors influencing fathers’ experiences need to be identified. According to Belsky (1984), parent personality is of central importance for parenthood and differences in personality influence variations in parenting (Prinzie et al., 2009). Implicit motives as part of one’s personality are likely to shape fathers’ subjective perceptions of fatherhood such as the degree of constraint he experiences due to his paternal role.

Research emphasizes the role of implicit motivation in our everyday lives due to its influence on social relationships. The implicit motivational system develops in early, pre-verbal childhood and is formed through emotional experiences (McClelland, 1987; Thrash et al., 2012). Implicit motives are defined as enduring preferences for specific types of incentives that are experienced as rewarding (Schultheiss, 2008). They are largely inaccessible to consciousness (McClelland et al., 1989). Nevertheless, they predict spontaneous and long-term behavioral trends by selecting, energizing, and orienting behavior toward preferred stimuli (McClelland et al., 1989). They influence cognitive and emotional processes and, as a consequence, social relationships (McClelland, 1987; Schultheiss and Brunstein, 1999). Motive satisfaction is achieved by engaging in motive-congruent behavior, meaning pursuing activities that are in line with our implicit motives (McAdams and Bryant, 1987). Since implicit motives represent the *need* for particular affective experiences, motive satisfaction leads to positive affect and higher emotional well-being (McClelland, 1985; Brunstein et al., 1995). Motive frustration, however, constitutes a hidden stressor and is associated with negative feelings (Hofer and Busch, 2011). According to McClelland’s Motive Disposition Theory (McClelland, 1985), the strength of an implicit motive determines the capacity for motive satisfaction or frustration. Thus, a high implicit motive results in either more positive or negative affect, depending on the ability to show corresponding behavior (McAdams and Bryant, 1987).

From an evolutionary perspective, motives increase our chances of survival and reproductive success (Baumeister and Leary, 1995; Buss, 2001). Several attempts to identify the most relevant motives for human behavior have been made, but no consensus has been reached so far (Aunger and Curtis, 2013). Interpersonal behavior, however, has consistently been described along the two dimensions of communion and agency or affiliation and power, respectively (Bakan, 1966; Brunstein et al., 1998; Stanton et al., 2010).

The implicit need for affiliation reflects a concern for establishing, maintaining, or restoring relationships (Schultheiss, 2008). Forming relational bonds was evolutionarily desirable since it increased chances of survival through the protection within a social network (Hall et al., 2010). Highly affiliation motivated individuals were more sympathetic toward others, showed more social behavior day by day and reported more positive affect when interacting with others (McAdams and Constantian, 1983; Sorrentino and Field, 1986; Koestner and McClelland, 1992).

The implicit motive for power is defined as a concern for having impact, influence, or control on others (Schultheiss, 2008). Ranking high in the group hierarchy and being in control over resources was an important prerequisite to ensure survival (Hall et al., 2010). As shown from a variety of studies, a high implicit power motive was associated with aggressive behavior (Winter, 1973; Mason and Blankenship, 1987), less relationship satisfaction (Stewart and Rubin, 1976), and a higher divorce rate (McClelland et al., 1972). However, the implicit power motive can also be enacted in a prosocial manner by teaching or providing unsolicited help (McClelland, 1975).

The influence of implicit motives in romantic or companionate relationships has been studied intensively. However, research about the role of implicit motives in fatherhood is rare, even though a lot of men become fathers or adopt a paternal role at some point in their lives (Martinez et al., 2012). Chasiotis et al. (2006) reported the implicit prosocial power motive to be positively related to the explicit love for children. The latter, in turn, was a significant predictor of becoming a parent. Peterson and Stewart (1993) found affiliation to be significantly related to the number of children and the amount of parenting involvement, but in women only. Interestingly, they also found a significant interaction of both affiliation and power motive. In women, a concurrence of low affiliation and low power motive was associated with less interest in parenting involvement. Several studies on the influence of implicit motives did not investigate parenthood directly but rather measured the degree of generativity, an important developmental life stage (Erikson, 1963). Generativity can be defined as a concern in establishing and guiding the next generation (Erikson, 1963). Therefore, becoming a parent is an important way to promote generativity (Snarey et al., 1987). McAdams et al. (1986) interviewed participants and had them write future-oriented texts. Power and intimacy motivation in participants’ texts were positively correlated with the amount of generative content in the interviews. Furthermore, implicit prosocial power motivation was associated with more generative concern, which was directly linked to higher global life satisfaction (Hofer et al., 2008).

However, not only the willingness to take over a paternal role should be considered but also the way men experience fatherhood. Father’s subjective view of their parental role could influence their parental involvement as well as their own health and well-being. Parenting is a challenging task and includes varying amounts of effort. A few decades ago, fulfilling the role as the family’s breadwinner was a father’s main responsibility (Goldscheider and Waite, 1991). Nowadays, fathers are expected not only to provide sufficient financial income but also to be actively engaged in family life and highly involved in childcare (Henwood and Procter, 2003). The demands of fatherhood can interfere with individual requirements such as work-related or social activities besides family life. As a consequence, fatherhood has been shown to have high impact on men, both in terms of benefits and costs such as increased work-family conflicts (Evenson and Simon, 2005; Zimmermann and Easterlin, 2006; Nomaguchi, 2009; Aassve et al., 2012; Nelson et al., 2013). According to the Role Strain Theory (Goode, 1960), occupying multiple and competing roles as employee, spouse, and father causes strain and has been associated with poorer health outcomes (van Hooff et al., 2005; Sekine et al., 2006). Moreover, fathers report more financial restrictions and constraints in social life (Claxton and Perry-Jenkins, 2008; Stanca, 2012; Wrzus et al., 2013). These constraints due to parenthood may increase psychological distress and may provoke psychopathological maladjustment such as depressive mood (Evenson and Simon, 2005; Umberson et al., 2010). They can also be harmful for parents’ romantic relationship resulting in lower marital satisfaction (Twenge et al., 2003).

Nevertheless, fatherhood is also regarded as beneficial. Several studies reported associations between parenthood and positive outcomes such as higher life satisfaction and overall well-being (Pleck, 1997; Eggebeen and Knoester, 2001; Nomaguchi and Milkie, 2003; Nelson et al., 2013). According to Role Enhancement Perspective, an individual’s role is a source of meaning and purpose (Simon, 1997; Burton, 1998) and occupying multiple roles provides benefits in terms of emotional gratification, increased resources, and higher psychological well-being (Sieber, 1974; Ahrens and Ryff, 2006). Correspondingly, fatherhood had a potentially buffering effect against work-related stress (Barnett et al., 1992; Haar and Bardoel, 2008). Moreover, holding a strong parental role identity and having positive role perceptions were related to the amount and quality of parenting behavior (Stone and McKenry, 1998; Rane and McBride, 2000; Bronte-Tinkew et al., 2006). In conclusion, occupying a paternal role is accompanied by varying degrees of perceived constraint as well as fulfillment and in turn, has negative or positive consequences for men’s well-being.

A number of factors influence parenting behavior and the individual adaptation to a positive father role. Belsky (1984) assumed that fatherhood is affected not only by contextual factors, such as marriage status, employment situation, or living conditions, but also by child and father characteristics. The demands of parenting young children may outweigh the rewards associated with parenthood (Umberson et al., 2010; Nelson et al., 2013). Particularly during the time of co-residing with young children, parents reported lower psychological well-being (Evenson and Simon, 2005). Similarly, parental age was found to be a moderator of the relationship between parenthood and life satisfaction. Whereas parents younger than 25 years were less satisfied with their lives compared to the same age group without children, parents aged 26–62 years reported significantly higher life satisfaction than their childless counterparts (Nelson et al., 2013). Parents aged 60 years or above reported less depression compared to childless adults of the same age (Chou and Chi, 2004). Working hours were found to be negatively associated with father involvement (Roeters et al., 2009), whereas highly educated fathers spent significantly more time with their children compared to less educated fathers (Yeung et al., 2001). However, parents with a high socio-economic status (SES) experienced fewer subjective benefits and less fulfillment in parenthood compared to parents with a low SES (Veroff et al., 1981; Kushlev et al., 2012).

Up to now, paternal involvement and well-being have been investigated in the context of sociodemographic characteristics, whereas little is known on the role of a man’s subjective experience of his paternal role as being constraining or fulfilling. Implicit motives as part of men’s personality are likely to be associated with their perception of fatherhood. The father-child relationship may provide incentives that are rewarding for men with certain motives.

### Present Research

This study aims to explore the role of implicit motives in fatherhood. *Perceived constraint due to fatherhood* measures the degree to which a father experiences negative consequences of his paternal role. We assume that a father’s perceived constraint is influenced by implicit motives since they affect subjective experiences, such as perception, cognition, and affect (McClelland et al., 1989; Deaux and Snyder, 2012). Individuals with a high need for affiliation derive pleasure from engaging in relationships such as the one between a father and his child (Schultheiss, 2008). Taking care of a child or children could be a way to satisfy a father’s need for affiliation. Therefore, we hypothesize that highly affiliation motivated fathers perceive less constraint, resulting in higher life satisfaction.

Peterson and Stewart (1993) argued that power motivation could be relevant in parenthood, since the parent-child relationship is authoritative rather than affiliative in nature. Moreover, fatherhood is a form of generativity (Snarey et al., 1987), which has consistently been linked to implicit power motivation. Therefore, we also expect to find less perceived constraint and consequently higher life satisfaction for fathers with a strong implicit need for power.

## Materials and Methods

We collected data as part of a larger cross-sectional project on the costs and benefits of fatherhood across lifespan. We conducted the project within the Central European Network on Fatherhood (CENOF). In the following, we describe only procedures and measures used for the present study. For more detailed information about the larger cross-sectional project see Waldvogel and Ehlert (2016).

### Participants and Procedure

Participants for the larger project were recruited in 2014 in all German-speaking countries of Middle Europe. Recruitment process included broadcast and newspaper announcements, online advertisements, mailing lists, and flyer distribution. Inclusion criteria for participation were male sex, age of 18 years or older, and occupying a paternal role for a child. Participants did not receive financial remuneration. Instead, four vouchers for leisure activities were drawn among all participants. The local Ethics Committee of the Faculty of Arts at the University of Zurich approved the study protocol before data collection. All participants provided an online informed consent. Data collection was divided into two parts and carried out online with respect to anonymity. First, we obtained sociodemographic information (e.g., father’s education) as well as psychometric and health data. Additionally, participants answered questions about their children (e.g., child’s age), their involvement in childcare (e.g., amount of time spent with children), and attitude toward fatherhood (e.g., perceived constraint). After completion of the first online survey, men were asked for their participation in the second part of the study and invitations were sent upon agreement. A total of 560 men participated in the online assessment of implicit motives. Participants were debriefed about the purpose of the motive assessment after its completion and were asked for their consent to use the data.

The present sample consisted of fathers (*N* = 276) who had biological children with one child mother, exclusively. All participants were in a stable relationship with the child’s mother. In addition, the couple lived in the same household as at least their youngest child. Participants had an average age of 39.34 years (*SD* = 6.68) with 72% having Swiss, 16% German, and 9% Austrian citizenship. Sixty-three percent of the sample had undergone tertiary education. Only 9% of the fathers were employed part-time.

### Measures

#### Implicit Motives

The Picture Story Exercise (PSE) is a standard story-writing instrument to measure participants’ implicit motive dispositions (Schultheiss and Pang, 2007). For this study, participants were shown six commonly used pictures to arouse implicit motives (Smith, 1992; Pang and Schultheiss, 2005). Each picture depicted a social situation with several characters involved. The pictures used were: boxer (McClelland and Steele, 1972), couple by river, ship captain, trapeze artists, women in laboratory (Smith, 1992), and nightclub scene (McClelland, 1975). We presented each picture for 10 s in a random order. After each picture, participants had 4 min to write an imaginative story about the scene depicted. Standard instructions and procedures were used as described in Smith (1992). The PSE could be run on any computer with an Internet connection. Measurements derived from online and paper-and-pencil versions of the PSE offer comparable results (Schultheiss et al., 2008; Bernecker and Job, 2011). We simultaneously coded stories for themes of affiliation (*n*Aff) and power (*n*Pow) using the Manual for Scoring Motive Imagery in Running Text (Winter, 1994, Unpulished). Affiliation imagery is defined as a concern with establishing, maintaining, or restoring friendly relations and was scored whenever a character expresses positive intimate feelings toward others, shows sadness about a separation, engages in affiliative activities, or nurturing friendly acts. Power imagery is coded whenever a character shows a concern with having impact or influence on others. It is scored whenever a character engages in strong, forceful actions, arouses an emotional reaction in others, provides unsolicited help or support, or tries to control, influence, or impress others. Two independent coders scored each story without knowledge about participants’ other characteristics. Both coders previously received training for coding motive content in written text. Each sentence was coded for motive imagery. Following recommendations by Schultheiss and Pang (2007), coders had several hours of scoring practice and have established at least 85% of inter-rater agreement on expert codings of calibration materials provided by Schultheiss (2015. Unpublished). Inter-rater reliability was estimated by Pearson correlations and was *r* = 0.91 for affiliation and *r* = 0.81 for power. We averaged scores for further analyses.

On average, participants wrote 486 words (*SD* = 129). Since story length was significantly correlated with the amount of motive imagery (*r* ≥ 0.40, *p* = 0.000), we used regression analyses to residualize motive scores for word count in order to remove the influence of verbal fluency (Schultheiss and Pang, 2007). We converted residuals to *z*-scores and used them in subsequent data analyses.

#### Perceived Constraint

Fathers rated the degree of perceived constraint due to fatherhood on a visual analog scale ranging from 0 (“not at all”) to 100 (“very”). Specifically, they were asked “How much do you feel that fatherhood constrains you?”

#### Life Satisfaction

In order to assess life satisfaction, participants answered the question “In general, how satisfied are you with your life?” They rated their degree of life satisfaction on a visual analog scale with 0 indicating “not satisfied at all” and 100 indicating “very satisfied.” Previous research showed that single-item measures of life satisfaction have acceptable reliability (Lucas and Donnellan, 2012).

### Statistical Analyses

Statistical analyses included several steps and were performed using the IBM Statistical Package for the Social Sciences (SPSS Version 22 for Windows). Statistical significance was defined as *p* < 0.05. We tested possible control variables estimated from literature for associations with main study variables by analyzing correlations. Paternal employment status and education were significantly correlated with life satisfaction (*r* = 0.14, *p* = 0.02) and perceived constraint (*r* = 0.12, *p* = 0.04), respectively. Perceived constraint was further related to age of child (*r* = -0.16, *p* = 0.01) which in turn was highly correlated with age of father (*r* = -0.71, *p* = 0.000). Therefore, we entered these control variables in all further analyses.

First, partial correlations between the main independent and dependent variables were computed, controlling for the above mentioned variables. Next, mediation analyses were computed to investigate the mediating effect of perceived constraint in the relationship between the predictors (*n*Aff and *n*Pow) and the outcome (life satisfaction). There are different statistical methods to test for mediation. The causal steps approach by Baron and Kenny (1986) has been widely used. However, it has been criticized in recent years due to several reasons such as lacking statistical power (MacKinnon et al., 2002; Hayes, 2009). Instead, Preacher and Hayes (2008) suggest using a bootstrapping approach to test for mediation. Especially for small to moderate sample sizes (*N* < 400) bootstrapping is suggested to have the greatest statistical power to detect mediation (McCartney et al., 2006). Furthermore, Preacher and Hayes (2004) argue that there still might be indirect effects despite the non-significance of the relationship between predictor and outcome which is the case for the present data. We used the SPSS script provided by Hayes (2015) to run the mediation analyses. We set bootstrapping to *k* = 10000 and computed 95% confidence intervals.

In a final step, we used the AMOS 23.0 software package to run structural equation modeling. Again, bootstrapping was set to *k* = 10000 and 95% bias-corrected bootstrap confidence intervals were computed (Preacher and Hayes, 2008). We applied a maximum-likelihood-technique and assessed the model fit using the χ^2^ statistic and other commonly used model fit indexes. In this study, a model was considered to have a good fit if all path coefficients were significant at the level of *p* < 0.05, χ^2^*/df* was < 2.5 (Bollen, 1989), RMSEA ≤ 0.05 (Steiger, 1990), and SRMR < 0.08 (Hu and Bentler, 1999). TLI and IFI indicate good model fits if they exceed 0.9 (Bollen, 1989; Hu and Bentler, 1999).

## Results

### Partial Correlations

Descriptives and inter-correlations among the main study variables are shown in **Table 1**. Perceived constraint was negatively correlated with *n*Aff (*r* = -0.14, *p* = 0.03) and life satisfaction (*r* = -0.24, *p* = 0.000) and showed a positive, significant relationship with *n*Pow (*r* = 0.13, *p* = 0.04).

**Table 1:** Descriptive statistics and inter-correlations among the relevant variables.

	Mean	*SD*	1	2	3	4
(1) Life satisfaction	80.83	13.61	1			
(2) Perceived constraint	41.15	27.34	-0.24^∗∗^	1		
(3) Affiliation (*n*Aff)^a^	7.33	3.23	0.06	-0.14^∗^	1	
(4) Power (*n*Pow)^a^	3.81	2.14	0.01	0.13^∗^	-0.07	1

*N = 276. Control variables: child’s age, paternal age, paternal education, paternal employment. SD, standard deviation. ^a^Raw motive scores are presented for descriptive statistics. Significance levels (two-tailed): ^∗^*p* < 0.05, ^∗∗∗^*p* < 0.001.*

### Mediation Analyses

Next, we were interested in the mediating effect of perceived constraint in the relationship between the predictors (*n*Aff and *n*Pow) and the outcome (life satisfaction). Age of child and father as well as paternal education and employment status were entered as control variables. Mediation analyses confirmed the significant relationships between predictors and mediator and between mediator and outcome. Moreover, they provided evidence for the mediating role of perceived constraint. The regression of life satisfaction on *n*Aff was not significant, *b* = 0.86, *t*(270) = 1.08, *p* = 0.28. The regression of the mediator perceived constraint on *n*Aff was significant, *b* = -3.65, *t*(270) = -2.24, *p* = 0.03. The mediator perceived constraint, controlling for *n*Aff, was significantly related to life satisfaction, *b* = -0.12, *t*(269) = -3.97, *p* = 0.000. *n*Aff, controlling for the mediator, was not a significant predictor of life satisfaction, *b* = 0.42, *t*(269) = 0.61, *p* = 0.61. The bootstrapped unstandardized indirect effect was *b* = 0.44. The 95% confidence interval ranging from 0.10 to 0.96 did not include zero, thus the indirect effect was statistically significant and mediation can be assumed (Preacher and Hayes, 2004).

The regression of life satisfaction on *n*Pow was not significant, *b* = 0.17, *t*(270) = 0.19, *p* = 0.85. The regression of the mediator perceived constraint on *n*Pow was significant, *b* = 3.41, *t*(270) = 2.11, *p* = 0.04. The mediator perceived constraint, controlling for *n*Pow, was significantly related to life satisfaction, *b* = -0.13, *t*(269) = -4.14, *p* = 0.000. *n*Pow, controlling for the mediator, was not a significant predictor of life satisfaction, *b* = 0.59, *t*(269) = 0.74, *p* = 0.46. The indirect effect was *b* = -0.43, 95% *CI* [-0.99, -0.07], giving evidence for the mediating role of perceived constraint.

### Structural Equation Modeling

**FIGURE 1:**
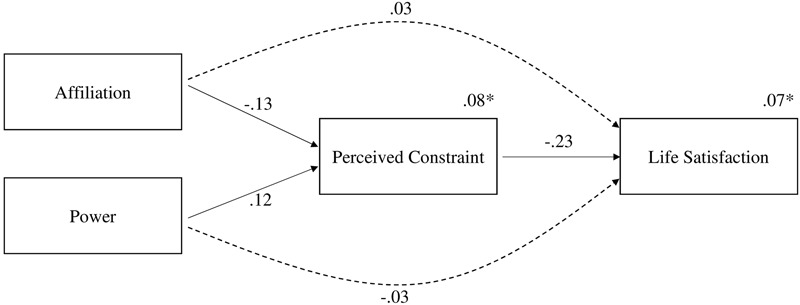
**Structural equation model showing direct and indirect effects of implicit motives on perceived constraint and life satisfaction.** Solid arrows indicate direct effects. Dashed arrows show indirect effects of affiliation and power on life satisfaction through the mediator perceived constraint. Numbers adjacent to arrows are standardized regression coefficients (*p* < 0.05). Control variables (not shown): child’s age, paternal education, paternal employment. ^∗^Variance explained by predictors.

In order to test the indirect effects of affiliation and power on life satisfaction via perceived constraint in an overall model, we applied structural equation modeling. Our model consisted of one endogenous variable (life satisfaction) and three exogenous variables (*n*Aff, *n*Pow, and perceived constraint), controlling for the previously mentioned variables. Since a father’s employment was associated with life satisfaction only, we modeled a single direct path from employment to life satisfaction. For child’s age and paternal education only direct paths to perceived constraint were allowed, because the relationship with life satisfaction was not significant. Paternal age was not significantly related to any outcome. Moreover, we encountered the problem of multicollinearity (Grewal et al., 2004), since paternal age was highly correlated with child’s age (*r* = 0.71, *p* = 0.000). Therefore, we modeled a second structural equation model without paternal age. Excluding paternal age from analyses showed no substantial drop in model fit indices. The final model is shown in **Figure 1**. The results showed that the model had a very good fit to the data: χ^2^ (5, *N* = 276) = 2.238, *p* = 0.815; χ^2^*/df =* 0.448; RMSEA = 0.000 (0.000, 0.051); SRMR = 0.015; TLI = 1.335; and IFI = 1.055.

Direct and indirect effects are shown in **Table 2**. The indirect effect of affiliation on life satisfaction was *b*_stand_ = 0.030 (0.007, 0.067), *p* = 0.01. In the same model, the indirect effect of power on life satisfaction was *b*_stand_ = -0.027 (-0.065, -0.003), *p* = 0.03. The standardized regression weights indicate small to medium effect sizes within our model (Cohen, 1990). The overall model accounted for 8% variance in perceived constraint and 7% variance in life satisfaction.

**Table 2:** Direct and indirect effects and 95% confidence intervals for the overall structural equation model.

			95% CI		
	*B*	*SE*	Lower bound	Upper bound	*p*
**Direct effects:**					
AFF → PC	-0.131	0.051	-0.232	-0.034	0.008
POW → PC	0.115	0.057	-0.002	-0.225	0.047
PC → LS	-0.232	0.063	-0.349	-0.105	0.000
**Indirect effects:**					
AFF → PC → LS	0.030	0.015	-0.007	-0.067	0.006
POW → PC → LS	-0.027	0.015	-0.065	-0.003	0.030

*N = 276. Control variables: child’s age, paternal education, paternal employment. AFF, Implicit affiliation motive; POW, Implicit power motive; PC, Perceived constraint; LS, Life satisfaction.*

## Discussion

### Summary of Results

The purpose of the present study was to investigate the influence of implicit motives on men’s perceived constraint due to fatherhood and their effect on men’s life satisfaction. Our study was the first to show that the implicit motives for affiliation and power have a significant direct impact on men’s subjective experiences in fatherhood. Moreover, perceived constraint acted as mediator in the relationship between fathers’ implicit motives and their satisfaction with life. As assumed in our first hypothesis, the implicit motive for affiliation was negatively related to perceived constraint. Therefore, fathers with a stronger need for affiliation reported being less constrained due to their paternal role. This, in turn, led to more life satisfaction. Our second hypothesis stated a negative relationship between implicit power motivation and perceived constraint. Interestingly, the results showed a positive association such that fathers with a high implicit power motive reported more perceived constraint, which lowered their life satisfaction.

### Interpretation of Results

The present findings can be embedded in previous research. Showing affiliative behavior will result in positive affect in individuals motivated for affiliation since they achieve motive satisfaction (McAdams and Bryant, 1987; Brunstein, 2006). The father-child relationship seems to provide incentives which are affectively desirable for affiliation motivated men since they can satisfy their motive within this relationship. In line with the Motive Disposition Theory (McClelland, 1985), a stronger motivation provides more capacity for motive satisfaction. Therefore, if highly affiliation motivated men are provided with opportunities to satisfy their implicit motivation in fatherhood they experience more positive affect and less constraint. It has to be further analyzed which kind of incentives within the father-child relationship might be particularly rewarding for high *n*Aff men such as traditional caregiving or playing.

Our results suggest that affiliation motivation potentially buffers against the negative consequences of fatherhood. These findings can be linked to research on the hormonal basis of social behavior. The hormone progesterone was positively related to both social bonding and affiliative behavior as well as implicit affiliation motivation (Schultheiss et al., 2003, 2004; Wirth and Schultheiss, 2006; Brown et al., 2009; Mehta and Josephs, 2011; Edelstein et al., 2014). Schultheiss et al. (2004) found increases in salivary progesterone levels after participants watched a movie, which aroused implicit affiliation. Therefore, implicit affiliation could be related to higher baseline levels of progesterone in fathers as well. Furthermore, engaging in motive-congruent affiliative behavior such as child interaction should then not only induce positive affect (McClelland, 1985) but also increase fathers’ progesterone levels and therefore may strengthen the father-child relationship. However, these assumptions still need to be tested in future studies.

Our hypothesis regarding the influence of implicit power on men’s perception of fatherhood was not supported by the present results. In our study we assessed general implicit power motivation. Since *n*Pow can be realized not only in an antisocial, more dominant way but also in a prosocial manner (McClelland, 1975), it could be important to differentiate the two domains. The few studies which investigated implicit power motivation in the context of parenthood or generativity either used a general measure of *n*Pow (McAdams et al., 1986; Peterson and Stewart, 1993) or concentrated specifically on implicit prosocial power motivation (Chasiotis et al., 2006; Hofer et al., 2008). Consequently, fatherhood might not provide the incentives that are affectively rewarding for high *n*Pow men, as the dominant aspects of implicit power are best satisfied in a hierarchical relationship structure. These hierarchical structures, for example, more often occur at the work place. Furthermore, the time and energy invested in the paternal role decreases the investment of both resources for men’s role at work. Thus, even fewer possibilities for power motive satisfaction are available, resulting in negative affect (McClelland, 1985). Following this interpretation, it is unsurprising that highly power motivated men feel more constraints due to their paternal role than fathers low in implicit power.

Our results can also be linked to studies on the hormone testosterone. Research assumes that social bonding suppresses testosterone to ensure sensitive parental behavior (Mazur and Michalek, 1998; Mehta and Josephs, 2011; van Anders et al., 2011). Implicit power motivation, however, was positively related to testosterone levels (Schultheiss et al., 2004; Schultheiss, 2007) and power motive satisfaction lead to an increase in testosterone (Schultheiss et al., 2005). Therefore, a high implicit power motive in fathers might be related to higher testosterone levels or higher testosterone reactivity. This in turn, might have adverse effects on nurturing parenting behavior, resulting in more perceived constraint.

The influence of both implicit affiliation and power motive on perceived constraint can be discussed within Role Identity Theory. The impact of occupying multiple roles on an individual’s well-being is still unclear (Ahrens and Ryff, 2006; Sekine et al., 2006). Apart from the quantity of roles, the salience or importance of each role for a person has to be considered. Rane and McBride (2000) had fathers rate how central the nurturing role was for their sense of self. Fathers who reported higher centrality were significantly more involved with their children. Moreover, health and well-being is positively influenced by strong and positive role perceptions (Pleck, 1985; Martire et al., 2000). Applying these results to the subject of the present study, we assume that fathers with a high implicit motive for affiliation should also perceive and value their paternal role as more important for their identity since engaging in this role is satisfying for their underlying motivational needs. This positive role evaluation could have led to a more positive perception of fatherhood.

However, this may not apply to highly power motivated individuals. Men with a high implicit dominant power motive may strongly identify with their role as employee or employer, which ideally gives them diverse opportunities to engage in power-oriented behavior. Conversely, their evaluations of their paternal role might be less positive compared to their employee or employer role. Especially since it diminishes resources that could have previously been invested in their role at work. Nevertheless, occupying the roles as a parent and worker might not be generally responsible for worse outcomes. Jutras and Veilleux (1991) found that men who combined the role of caregiver with other family roles experienced a higher subjective burden than women who combined these roles. Accordingly, research might have to distinguish the different role combinations as it could be important which roles are occupied at the same time. High *n*Pow men might not experience role strain as long as the power motive is satisfied within any of their roles.

Our findings on the influence of subjective experiences on life satisfaction are in accordance with research showing that the positive effects of parenthood on life satisfaction might be overshadowed by the costs of children, both in terms of finances and time (Pollmann-Schult, 2014). Fathers who were highly motivated for power seem to have experienced increased costs due to their paternal role, which led to lower life satisfaction. Implicit affiliation, however, had a buffering effect against the negative experiences in fatherhood and fathers were then more satisfied with their lives. Concluding, we were able to demonstrate different influences of affiliation and power motivation on men’s perceptions of their paternal role and their well-being.

### Limitations and Future Research

Several limitations of this study should be discussed. First, due to the cross-sectional design of the study, our results cannot be interpreted in terms of causal directionality. However, the presumed causal directions are the most theoretically plausible. Nevertheless, we cannot rule out the possibility that men who enter parenthood differ from men who remain childless in regard to the strength of their implicit motives. For example, men with a high implicit motive for affiliation seek relationships and might be more willing to become a father in order to establish a deeper bond to their partner as well as a new relationship to their child. Alternatively, the transition to parenthood and the child’s or children’s upbringing might change a man’s implicit motives since they are shaped by strong emotional experiences. Longitudinal studies are needed to compare fathers to non-fathers over lifespan and to investigate if highly emotional life events, such as becoming a parent, alter men’s implicit motives.

A second limitation concerns the measurement methods. For both perceived constraint and life satisfaction we used a single visual analog scale. This has proven to be a valid measure for life satisfaction (Lucas and Donnellan, 2012). However, we observed a ceiling effect in our data, probably due to a selection or social desirability bias. Future studies could consider using measures which consist of several items such as the Satisfaction with Life Scale (Diener et al., 1985). To our knowledge, this was the first study to apply this type of measure for perceived constraint. Most previous studies on parental experiences were qualitative in nature and conducted interviews with participants (e.g., Baruch and Barnett, 1986; Silverstein et al., 1999; Maridaki-Kassotaki, 2000; Blair-Loy, 2003). Not only was our single measure more time and cost efficient but, due to our anonymous online survey, we could also reduce the risk of a socially desirable response pattern. Studies have consistently shown that face-to-face interviews increase the probability of socially desirable responses compared to self completion measurements (Richman et al., 1999; Tourangeau and Yan, 2007). In a meta-analysis, computerized surveys as applied for the present study produced the most truthful responses (Gnambs and Kaspar, 2015). It could be informative, though, to add further items which assess different types of constraint (e.g., financial or time constraint) in order to receive a more comprehensive measure of perceived constraint (e.g., Scott and Alwin, 1989). Furthermore, future research may want to add objective constraint, such as assessments from independent observers, as a control variable. Nevertheless, research shows that subjective ratings are relevant in predicting outcomes (DeSalvo et al., 2006; Rasmussen et al., 2009). Therefore, we encourage the use of subjective measurements in future research.

As previously mentioned, the PSE applied in this study revealed the strength of a general implicit power motive only. However, power motivation can be enacted in a more dominant, aggressive way or in a prosocial manner (McClelland, 1975). Different results might be obtained by distinguishing these two components of implicit power motivation. However, *post hoc* analyses with the present data revealed no significant results for individual subcategories of implicit power motivation. Furthermore, while both implicit affiliation and power motive had an independent effect on perceived constraint, their interaction effect yielded no significant result. Nevertheless, future studies might still consider analyzing these different aspects of implicit motivation and their relation to fathers’ perceived constraint.

Finally, the homogeneity of the analyzed sample has to be taken into account. We exclusively focused on biological fathers in traditional family structures. However, alternative and more complex family structures have emerged in the past years (Parke, 2013). It is unclear to which degree our results can be generalized for other types of modern fatherhood such as social, adoptive, or step fathers.

## Conclusion

This is the first study showing that implicit motives shape men’s experiences in fatherhood. Implicit affiliation motivation had a buffering effect on fathers’ perceived constraint and a positive indirect influence on their life satisfaction. In contrast, men who had a stronger implicit need for power reported a higher degree of constraint due to fatherhood and were less satisfied with their lives in general. Our study highlights the importance of providing congruent motivational incentives for an individual’s well-being. As a practical implication, finding opportunities to satisfy men’s implicit motives could improve their subjective experiences in fatherhood and their well-being.

## Author Contributions

PW was involved in the planning of the study design and conducted the study at the University of Zurich. JR coded the stories for implicit motives. She also performed data analyses and prepared the manuscript as well as figures and tables. UE developed the study design and supervised the study including its preparation and execution and further revised the manuscript. All authors approved the final version of the manuscript for submission.

## Conflict of Interest Statement

The authors declare that the research was conducted in the absence of any commercial or financial relationships that could be construed as a potential conflict of interest.
